# Evolution of *Anolis* Lizard Dewlap Diversity

**DOI:** 10.1371/journal.pone.0000274

**Published:** 2007-03-07

**Authors:** Kirsten E. Nicholson, Luke J. Harmon, Jonathan B. Losos

**Affiliations:** 1 Department of Biology, Central Michigan University, Mt. Pleasant, Michigan, United States of America; 2 Biodiversity Centre, University of British Columbia, Vancouver, British Columbia, Canada; 3 Museum of Comparative Zoology and Department of Organismic and Evolutionary Biology, Harvard University, Cambridge, Massachusetts, United States of America; University of Exeter, Cornwall Campus, United Kingdom

## Abstract

**Background:**

The dewlaps of *Anolis* lizards provide a classic example of a complex signaling system whose function and evolution is poorly understood. Dewlaps are flaps of skin beneath the chin that are extended and combined with head and body movements for visual signals and displays. They exhibit extensive morphological variation and are one of two cladistic features uniting anoles, yet little is known regarding their function and evolution. We quantified the diversity of anole dewlaps, investigated whether dewlap morphology was informative regarding phylogenetic relationships, and tested two separate hypotheses: (A) similar *Anolis* habitat specialists possess similar dewlap configurations (Ecomorph Convergence hypothesis), and (B) sympatric species differ in their dewlap morphologies to a greater extent than expected by chance (Species Recognition hypothesis).

**Methodology/Principal Findings:**

We found that dewlap configurations (sizes, patterns and colors) exhibit substantial diversity, but that most are easily categorized into six patterns that incorporate one to three of 13 recognizable colors. Dewlap morphology is not phylogenetically informative and, like other features of anoles, exhibits convergence in configurations. We found no support for the Ecomorph Convergence hypothesis; species using the same structural habitat were no more similar in dewlap configuration than expected by chance. With one exception, all sympatric species in four communities differ in dewlap configuration. However, this provides only weak support for the Species Recognition hypothesis because, due to the great diversity in dewlap configurations observed across each island, few cases of sympatric species with identical dewlaps would be expected to co-occur by chance alone.

**Conclusions/Significance:**

Despite previous thought, most dewlaps exhibit easily characterizable patterns and colorations. Nevertheless, dewlap variation is extensive and explanations for the origin and evolution of this diversity are lacking. Our data do not support two hypothesized explanations for this diversity, but others such as sexual selection remain to be tested.

## Introduction

Animals convey information to one another through a broad variety of signaling mechanisms [1]–[3]. Communication may be necessary for territory establishment or defense, reproductive interactions, predator defense, or resource location. Communication systems vary between species and take a variety of forms including auditory and visual displays.

Lizard communication systems have evolved to form complex displays and can exhibit extraordinary diversity [4]–[6]. The complex displays of some lizards have been studied from a proximate behavioral or ecological point of view to identify the relevant components and contexts of the signals. To be effective, signals must evolve within the context of the environmental, perceptual, sexual, and social selection pressures facing a species [5]–[9]. One of the goals of comparative evolutionary biology is to identify the evolutionary forces responsible for generating this diversity.

**Figure 1:**
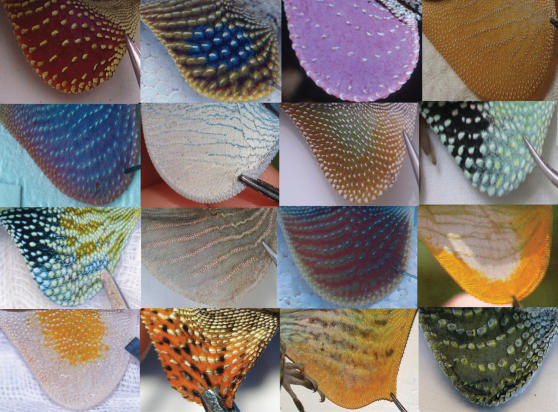
A small sample of *Anolis* dewlaps exemplifying observed morphological diversity. Some images are modified from original photographs and used with permission from David Hillis and Richard Glor. Species depicted are as follows (in order right to left and top to bottom): *A. pulchellus, A. sericeus, A. liogaster, A. longitibalis, A. cobanensis, A. gorgonae, A. cristatellus, A. chlorocyanus, A. reconditus, A. christophei, A. cuprinus, A. new species, A. lineatopus, A. annectens, A. baleatus, A. auratus*.

The dewlaps of *Anolis* lizards present a classic example of a complex signaling system whose function and evolution is poorly understood. A characteristic and charismatic feature of *Anolis*, the dewlap consists of a flap of skin below the chin that is supported by the second ceratobranchial cartilage, a modification of the hyoid apparatus [10]–[11]. Dewlaps vary dramatically in size, shape, color, and pattern (Figure 1), and are frequently used to delineate species boundaries (e.g., see 12 and references therein). Anoles extend and retract their dewlaps in various temporal patterns, frequently combined with head and other body movements, that are thought to communicate mating and territorial interests [4], [13], as well as being used in predator deterrence [14]. In addition, the cadence of head-bobbing used in these visual displays appears to be species specific [4 and references therein].

The breadth of morphological diversity demonstrated by *Anolis* dewlaps is impressive (Figure 1). This broad diversity gives the impression that no two dewlaps are exactly alike. While a few studies have examined the evolution of anole dewlaps [15]–[17], empirical characterization of dewlap diversity has so far never been attempted. Without such a survey, the extent to which dewlaps are unique cannot be assessed. In addition, systematists often use dewlap features as taxonomic characters, yet no test of the evolutionary lability of dewlaps has ever been conducted.

Explanations for the evolution of dewlap diversity are lacking, although two hypotheses may be relevant. The Ecomorph Convergence hypothesis [18] is based on the extensive convergence seen among the anole radiations in the Greater Antilles. On each island, anoles have radiated mostly independently, producing the same set of habitat specialists, termed “ecomorphs”, on each island [19]–[20]. Convergence among members of the same ecomorph class involves limb and tail length, head dimensions, toe-pad characteristics, sexual dimorphism, and other characters [18], [21]–[23]. We test whether this convergence extends to the configuration of the dewlap.

The Species Recognition hypothesis [17] predicts that sympatric species should evolve characteristics that aid in distinguishing conspecifics from heterospecifics. Application of this hypothesis to anoles predicts that sympatric species should have distinctly different dewlaps. This hypothesis has been examined [17] or discussed [24]–[30] in several studies that propose that dewlap colors have evolved to allow the rapid identification of heterospecifics.

The Ecomorph Convergence and Species Recognition hypotheses have not yet been tested extensively across anoles. Previous tests (discussed above) have focused on small communities or comparisons among small subsets of species. Considerable information is now available on the dewlap characteristics of most Caribbean anoles. This increase in available data allows the extension of previous studies that have focused upon individual components of dewlap morphology (color or size only). Importantly, phylogenetic relationships of Caribbean anoles are now well resolved [20], [31]–[33] allowing for the analysis of morphological data within a reliable phylogenetic context.

The goals of this study were therefore 1) to quantify the diversity in Caribbean *Anolis* dewlap morphologies, 2) examine the extent to which dewlap morphology contains phylogenetic signal, versus the alternative hypothesis that no relationship exists between degree of phylogenetic relatedness and dewlap similarity, and 3) test two separate hypotheses that may explain dewlap diversity: (A) do similar *Anolis* habitat specialists possess similar dewlap configurations (Ecomorph Convergence hypothesis), and (B) do sympatric species differ in their dewlap morphologies to a greater extent than expected by chance (Species Recognition hypothesis)? We have focused our study on Caribbean species because precise information on phylogenetic relationships and dewlap configurations are as yet incomplete for mainland taxa.

## Methods

We categorized three components of dewlap appearance: pattern, color, and size. We collected information on dewlap color and patterns from detailed published literature [12], [34]–[37], photographs taken in the field, and from experts familiar with these species (summarized in Table 1). Dewlap patterns were classified into categories (see Results for complete descriptions of all pattern categories). To categorize the proportion of each color present in a given pattern, we implemented a standardized approach in which we measured the area occupied by each component of the pattern on the dewlap of a single, representative species with that pattern. For example, a representative of a bicolored marginal dewlap was measured for the area comprising the margin (10%) and the remainder (90%) of the dewlap. All other species possessing this pattern were recorded as having 10%color A and 90%color B. In a few cases, components of species' patterns departed obviously from these standardized sizes, and were estimated accordingly. Descriptions in the literature and personal observations of dewlap colors did not follow any standard color scale [38]; therefore, all dewlaps were classified according to conventional color categories. Spectrophotometric data would most objectively represent dewlap colors, but these data are currently available for few species [e.g.], [25, 28]; although some workers are now collecting such data, it will likely be many years before a large data set will become available. Our approach, then, is preliminary; taking advantage of the wealth of information on dewlap colors currently available, while recognizing that eventually such data will be refined by the availability of more precise and standardized spectrophotometric data. Categorizing color data will tend to underestimate diversity by lumping dewlaps that actually differ spectrally into the same categories. As a result, these data will tend to bias our study to incorrectly detect convergent evolution. Data on dewlap sizes were taken from [16] which reported relative dewlap sizes as residuals of actual dewlap size regressed against snout-vent length. Hereafter we use the term “configuration” to refer to particular combinations of dewlap color(s), pattern and size.

**Table 1:** List of species included in this study and source of dewlap information (authors indicated by initials, * = Richard E. Glor).

Species	Citation	Pers. Obs.	Species	Citation	Pers. Obs.
*C. barbatus*	S&H 1991	JBL	A. homolechis	S&H 1991; LRS 1999	JBL
*C. chamaeleonides*	S&H 1991	JBL	A. imias	S&H 1991; LRS 1999	
*C. porcus*	S&H 1991	JBL	A. inexpectatus	S&H 1991; LRS 1999	
*“C'norops” barbouri*	S&H 1991; P&H 1996	JBL	A. insolitus	S&H 1991	JBL
*A. acutus*	S&H 1991	KEN	A. isolepis	S&H 1991; LRS 1999	JBL
*A. aeneus*	S&H 1991		A. juangundlachi	S&H 1991; LRS 1999	JBL
*A. ahli*	S&H 1991; LRS 1999		A. jubar	S&H 1991; LRS 1999	JBL
*A. alayoni*	LRS 1999		A. koopmani	S&H 1991	
*A. alfaroi*	LRS 1999		A. krugi	S&H 1991; Rivero 1978	JBL, *
*A. aliniger*	S&H 1991	JBL	A. lineatopus	S&H 1991	JBL
*A. allisoni*	S&H 1991; LRS 1999	JBL	A. lividus	S&H 1991	
*A. allogus*	S&H 1991; LRS 1999	JBL	A. longiceps	S&H 1991	
*A. altavelensis*	S&H 1991		A. longitibialis	S&H 1991	JBL, *
*A. alumina*	S&H 1991	JBL	A. loysiana	S&H 1991; LRS 1999	JBL
*A. alutaceus*	S&H 1991; LRS 1999	JBL	A. luciae	S&H 1991	
*A. anfilioquioi*	S&H 1991; LRS 1999		A. lucius	S&H 1991; LRS 1999	
*A. angusticeps*	S&H 1991; LRS 1999	JBL	A. luteogularis	S&H 1991; LRS 1999	JBL
*A. argenteolis*	S&H 1991; LRS 1999	JBL	A. macilentus	LRS 1999	
*A. argillaceus*	S&H 1991; LRS 1999		A. marcanoi	S&H 1991	JBL
*A. armouri*	S&H 1991		A. marmoratus	S&H 1991	
*A. bahorucoensis*	S&H 1991	JBL	A. marron	S&H 1991	
*A. baleatus*	S&H 1991	JBL	A. maynardi	S&H 1991	
*A. baracoe*	S&H 1991; LRS 1999		A. mestrei	S&H 1991; LRS 1999	JBL
*A. barahonae*	S&H 1991; P&H 1996	JBL	A. monensis	S&H 1991; Rivero 1978	
*A. bartschi*	S&H 1991; LRS 1999	JBL	A. monticola	S&H 1991	
*A. bimaculatus*	S&H 1991		A. noblei	S&H 1991; LRS 1999	
*A. bremeri*	S&H 1991; LRS 1999		A. nubilis	S&H 1991	
*A. brevirostris*	S&H 1991	JBL, *	A. occultus	S&H 1991; Rivero 1978	JBL
*A. brunneus*	S&H 1991		A. oculatus	S&H 1991	
*A. carolinensis*	Ashton and Ashton 1991	LJH, JBL, KEN, *	A. olssoni	S&H 1991	JBL
*A. caudalis*	S&H 1991		A. opalinus	S&H 1991	JBL
*A. centralis*	S&H 1991; LRS 1999	JBL	A. ophiolepis	S&H 1991; LRS 1999	
*A. chlorocyanus*	S&H 1991	JBL, KEN	A. paternus	S&H 1991; LRS 1999	
*A. christophei*	S&H 1991	JBL, *	A. pigmaequestris	S&H 1991; LRS 1999	
*A. clivicola*	S&H 1991; LRS 1999	*	A. pinchoti	S&H 1991	
*A. coelestinus*	S&H 1991	JBL, *	A. placidus	S&H 1991	JBL
*A. concolor*	S&H 1991		A. poncensis	S&H 1991; Rivero 1978	JBL
*A. confusus*	LRS 1999		A. porcatus	S&H 1991; LRS 1999	KEN
*A. conspersus*	S&H 1991; P&H 1996		A. pulchellus	S&H 1991; Rivero 1978	JBL, *
*A. cooki*	S&H 1991; Rivero 1978	JBL	A. pumilis	S&H 1991; LRS 1999	
*A. cristatellus*	S&H 1991; Rivero 1978	JBL, KEN	A. quadriocellifer	S&H 1991; LRS 1999	
*A. cupeyalensis*	S&H 1991; LRS 1999		A. reconditus	S&H 1991	
*A. cuvieri*	S&H 1991; Rivero 1978	JBL	A. richardi	S&H 1991	
*A. cyanopleurus*	S&H 1991; LRS 1999		A. ricordii	S&H 1991	JBL
*A. cybotes*	S&H 1991	JBL, KEN	A. rimarum	S&H 1991	
*A. darlingtoni*	S&H 1991		A. roosevelti	S&H 1991; Rivero 1978	
*A. delafuentei*	S&H 1991; LRS 1999		A. roquet	S&H 1991	
*A. desachensis*	S&H 1991		A. rubribarbus	S&H 1991; LRS 1999	
*A. distichus*	S&H 1991	JBL, KEN, *	A. rupinae	S&H 1991	
*A. dolichocephalus*	S&H 1991		A. sabanus	S&H 1991	
*A. equestris*	S&H 1991; LRS 1999	KEN	A. sagrei	S&H 1991; LRS 1999	LJH, JBL, KEN, *
*A. ernestwilliamsi*	S&H 1991		A. scriptus	S&H 1991	
*A. etheridgei*	S&H 1991	JBL	A. semilineatus	S&H 1991	JBL
*A. eugenegrahami*	S&H 1991		A. sheplani	S&H 1991	JBL
*A. evermanni*	S&H 1991; Rivero 1978	JBL	A. shrevei	S&H 1991	JBL
*A. extremus*	S&H 1991		A. singularis	S&H 1991	JBL
*A. fairchildi*	S&H 1991		A. smallwoodi	S&H 1991; LRS 1999	
*A. ferreus*	S&H 1991		A. smaragdinus	S&H 1991	
*A. fowleri*	S&H 1991	JBL	A. spectrum	S&H 1991; LRS 1999	
*A. fugitivus*	S&H 1991; LRS 1999		A. strahmi	S&H 1991	JBL
*A. garmani*	S&H 1991	JBL, KEN	A. stratulus	S&H 1991; Rivero 1978	JBL
*A. garridoi*	LRS 1999		A. stratulus	S&H 1991; Rivero 1978	
*A. gingivinus*	S&H 1991		A. trinitatus	S&H 1991	
*A. grahami*	S&H 1991	JBL	A. valencienni	S&H 1991	JBL
*A. griseus*	S&H 1991		A. vandicus	S&H 1991; LRS 1999	
*A. guafe*	LRS 1999		A. vermiculatus	S&H 1991; LRS 1999	JBL
*A. guazuma*	S&H 1991; LRS 1999		A. vescus	LRS 1999	
*A. gundlachi*	S&H 1991; Rivero 1978		A. wattsi	S&H 1991	
*A. haetianus*	S&H 1991		A. websteri	S&H 1991	
*A. hendersoni*	S&H 1991		A. whitemani	S&H 1991	JBL

Citation abbreviations are as follows: *S&H 1991:* [12] (and references therein); *P&H 1996*: [35]; *LRS 1999*: [37]; *Rivero 1978*: [36].

### Phylogenetic Signal

We tested whether any phylogenetic signal exists for each of the three categories of dewlap configuration (pattern, color, and size) within the context of a current anole phylogeny [20]. The Nicholson et al. [20] tree is based upon 1483 aligned base pairs of DNA sequences for 7 mitochondrial genes (partial COI, complete ND2 and five complete tRNA's) and reconstructed using both parsimony and Bayesian methods. We used their consensus Bayesian tree for our analyses, and rendered it ultrametric using the program r8s [39] so that branch lengths represented an index of time. Species were excluded using the program TreeEdit [40] if we lacked dewlap pattern information.

For this test, we created three morphological distance matrices representing dissimilarities in pattern, color, and size. The pattern matrix consisted of scores of pairwise similarities (0) or differences (1) depending on whether two species shared the same pattern. In cases of polymorphism, species were considered to have the same dewlap patterning if any morph of one species had the same pattern as a morph in the other species. Dewlap colors were represented in a matrix in which we recorded the proportion of dewlap area represented by each color (e.g., 4%of the dewlap area was red, 38%white, 58%blue) for each species. To calculate dewlap color dissimilarity between species, we calculated pairwise Manhattan distances. In this case we wanted to emphasize the absolute difference in dewlap coloration between pairs of species in terms of overall shared coloration (both number of colors as well as proportion of each color shared) which Manhattan distances represent better than Euclidean distances. The dewlap size matrix represented the difference in relative dewlap area between species. Data on dewlap sizes was taken from [16] and pairwise differences calculated.

We created a phylogenetic distance matrix with distances calculated on the ultrametric phylogenetic tree for these species. Using Mantel tests, we tested for a relationship between each morphological distance matrix and the phylogenetic distance matrix [41]. Significance was determined by comparing the matrix correlation statistic to a distribution obtained by permuting the matrices 9,999 times, using the program Permute! [42] and implementing the double permutation method option and backward elimination and forward selection testing options. Subsequent comparisons using Permute!, described below, follow the same procedure.

### Ecomorph Convergence Hypothesis

We tested the Ecomorph Convergence hypothesis, which predicts that *Anolis* species living in similar structural habitats will have similar dewlap configurations. Six structural habitat categories were recognized, corresponding to the different ecomorph classes (grass-bush, trunk-ground, trunk, trunk-crown, crown-giant, and twig) recognized by Williams [43]. Ecomorph designations were based on previous studies [e.g.], [19, 21] or on our unpublished observations. We used a multiple Mantel test [44] with four similarity matrices to test this hypothesis. The first matrix indicated whether pairs of species were in the same or different ecomorph categories. A matrix of ecomorphs was generated in which pairs of species in the same ecomorph category were given a score of 0, and pairs of species in different categories were given a score of 1. Three additional matrices representing patterns, colors, and sizes were generated using the same approach as in the previous paragraph.

To remove the effect of phylogenetic relatedness, we regressed ecomorphs, pattern, size, and color matrices onto the phylogenetic distance matrix, and retained the residuals for subsequent analysis. We then carried out multiple matrix regression using the program Permute! [42] with dewlap color, pattern, and size as dependent variables, and ecomorph as the independent variable. This hypothesis predicts that there will be a significant correspondence between dewlap configuration and ecomorph category.

### Species Recognition Hypothesis

The species recognition hypothesis predicts that sympatric species will have different dewlap configurations [16]. We focused on four communities of sympatric anoles (Soroa, Cuba–10 species, including *A. vermiculatus*, which does not have a dewlap; La Palma, Hispaniola–7 species [*A. cybotes* and *A. ricordii* not included because dewlap size data were not available]; Negril, Jamaica–5 species, not including *A. sagrei*, which is a recent colonist [45]; Luquillo Mountains, Puerto Rico–8 species). To test this hypothesis, we counted the number of identical dewlap pairs within each of the four *Anolis* communities. Dewlaps were considered identical if they had the same pattern, the same proportions of colors, and a residual dewlap size differing by less than 0.2 (other values for size similarity cutoffs were tried, but the results were not affected; data not shown). Because the dewlap of *Anolis cristatellus* (Puerto Rico) occurs in four polymorphic forms, we repeated the analysis separately with each of these forms.

We investigated whether dewlap similarity among sympatric species was less than would be expected if communities were composed of a random set of species. To this end, we generated 9,999 random communities by creating random communities with the same number of species (created by selecting without replacement from the pool of all species in these four communities). We dealt with polymorphic species by considering each form as a unique entity in the null pool. We then compared the number of identical species in each random community to the number from the actual community. We calculated a p-value for this test as (number of random communities with the same or fewer identical pairs+1)/10,000.

## Results

### Patterns and Colors

**Figure 2:**
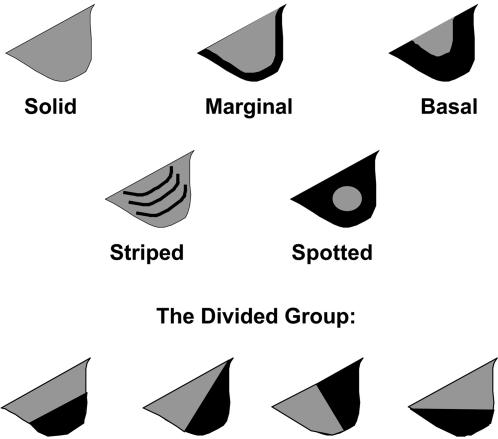
Dewlap patterns categorized by this study. Six patterns were observed among Caribbean *Anolis* species. While five of the patterns (Solid, Marginal, Basal, Striped, and Spotted) were observed with some frequency, four additional morphs were observed so rarely that they were grouped together within a sixth category entitled Divided.

Most dewlaps exhibited one of six patterns (Figure 2), although a few species had other patterns (Table 2). Solid dewlaps, which contain only one color across the entire dewlap surface, were far-and-away the most common pattern amongst Caribbean anoles (Table 2). Marginal dewlaps have a single color that covers most of the dewlap (∼90%) and a second color along the outer margin representing about 10%of the total area. Spotted dewlaps have a clear, circular spot in the center that covers roughly 10%of the total area. Basal dewlaps are similar to spotted dewlaps, except that the spot is clearly positioned at the base of the throat instead of in the middle of the dewlap, and generally comprises more surface area than does the spot in spotted dewlaps (∼12%area). Striped dewlaps may exhibit rows of scales of a strongly contrasting color to their background, or may be composed of differently colored skin and cover approximately 4–5%of the total area. Divided dewlaps included those that exhibited two color patches that each covered approximately 50%of the total area. These patches could be arranged dorsoventrally, anteroposteriorly or diagonally. In addition to these six categories, 12%of the species' dewlaps we refer to as Unique because they exhibit complex combinations of the above-mentioned patterns (e.g., marginal+striped; divided+marginal) or do not fit any of the categories described above (e.g., *A. marcanoi* and *A. scriptus* exhibit blotched or marbled dewlaps). Also, in a few (4%) species, dewlaps are completely absent (they lack the second ceratobranchial cartilage) or sufficiently reduced to be considered absent.

**Table 2:** Distribution of dewlap patterns exhibited by Caribbean anoles.

Pattern	Number	% of Exhibited Morphs	Colors
Solid	90	58.4%	13
Marginal	14	9.0%	12
Spotted	6	4.0%	8
Basal	4	2.6%	2
Chin	1	0.7%	2
Stripped	9	5.8%	10
Lateral	7	4.5%	12
Unique	17	11.0%	13
Absent	6	4.0%	

Data from 140 Caribbean species. The last column gives an indication of color diversity within each pattern category. *The number of exhibited patterns does not total 140 because polymorphic species exhibit more than one pattern.

Thirteen dewlap colors (red, yellow, green, greenish-yellow, blue, orange, black, white, peach, gray, pink, purple, brown) were observed (Table 3). Yellow, orange, and red are the most common colors in all pattern types (ranges from 42–83%depending on pattern category). Despite the diversity of colors exhibited among all patterns, only 65 combinations of patterns and colors were observed among anole dewlaps, much less than the 793 possible combinations (13 solid dewlaps+156 possible combinations of 2 out of 13 colors [78 combinations multiplied by two because each pair of colors can occur in two different arrangements depending on which color is the more common] * 5 dewlap patterns).

**Table 3:** Colors exhibited by each pattern.

Pattern	Color	Numbers
Solid	Yellow	39
	Orange	27
	Red	13
	White	10
	Pink	7
	Brown	7
	Green	6
	Greenish yellow	6
	Gray	5
	Peach	4
	Blue	3
	Black	2
	Purple	1
Marginal	Yellow	11
	Red	4
	Orange	5
	Greenish yellow	1
	Black	1
	White	2
	Gray	2
	Pink	1
Spotted	Red	5
	Yellow	4
	Orange	3
	Green	1
	Greenish yellow	1
	Blue	1
	Brown	1
Basal	Red	5
	Yellow	5
	Greenish yellow	1
	Purple	1
Striped	yellow	4
	White	4
	Orange	3
	Red	2
	Green	1
	Peach	2
	Gray	1
	Pink	1
	Purple	1
Lateral	Red	2
	Yellow	2
	Gray	2
	Orange	1
	Black	1
	Peach	1
	Pink	1

Numbers in the right-hand column refer to the number of morphs exhibiting the color in the indicated pattern, yielding an indication of color diversity within each pattern.

### Phylogenetic Signal

**Figure 3:**
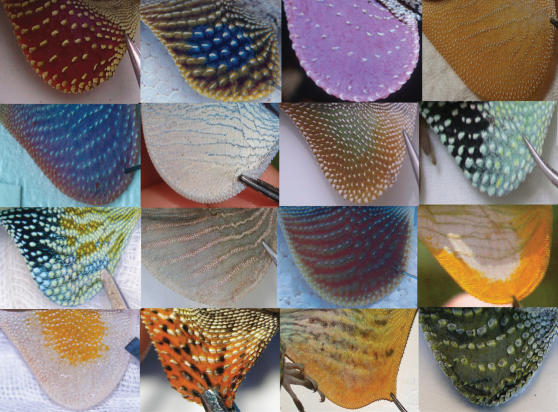
Dewlap patterns mapped on to a phylogeny for *Anolis* species. Patterns are indicated in color on the phylogeny (color legend upper left). Polymorphic species are those that exhibited two or more pattern morphs (see text for how this was handled analytically). Patterns are defined in the text. This tree includes all of the species used in the tests for phylogenetic signal of dewlap characters. The tree is modified from Nicholson et al.'s [20] anole tree but has been pruned of taxa for which dewlap information was lacking. Analyses were based on an ultrametric version of this tree, but is reproduced here in cladogram form for enhanced viewing of dewlap pattern information.

All species included in the tests for phylogenetic signal in dewlap morphology are shown in Figure 3. Dewlap patterns, colors, and sizes are not phylogenetically informative; that is, no relationship exists between how closely related two species are and how similar they are in any feature of the dewlap. Figure 3 depicts dewlap patterns on a phylogeny (colors and sizes not shown). Patterned dewlaps have clearly evolved multiple times from ancestors possessing solid dewlaps. However, the addition of color and/or size data (not shown on the tree) eliminates all monophyletic groups of anoles with identical dewlaps (i.e., not even a single pair of sister taxa have identical dewlap configurations). Mantel tests showed a lack of phylogenetic signal for each of the dewlap characters (pattern: R^2^ = 0.018, P = 0.13; color: R^2^ = 0.001, P = 0.63; size: R^2^ = 0.023, P = 0.07)

### Ecomorph Convergence Hypothesis

No support was observed for the hypothesis that *Anolis* lizards in the same structural habitat category are more similar in dewlap configuration than expected by chance (four-way Mantel test, overall R^2^ = 0.048; P = 0.21) (Table 4).

**Table 4:** Data used to test the Ecomorph Convergence hypothesis.

Species	Community	Ecomorph	Relative Size	Pattern	Color
*A. ahi*	Cuba	trunk-ground	0.958	Basal	yellow & red
*A. allisoni*	Cuba	trunk-crown	−0.423	Chin	pink & white
*A. allogus*	Cuba	trunk-ground	1.065	Striped	yellow & red
*A. alutaceus*	Cuba	grass-bush	0.396	Solid	yellow
*A. angusticeps*	Cuba	twig	−0.206	Solid	pink
*A. bartschi*	Cuba	unique	−1.635	–	–
*A. equestris*	Cuba	crown-giant	−0.385	Unique	pink, blue, & yellow
*A. guazuma*	Cuba	twig	−0.239	Solid	white
*A. homolechis*	Cuba	trunk-ground	0.71	Solid	white/gray
*A. loysiana*	Cuba	trunk	0.863	Unique	yellow & red
*A. lucius*	Cuba	unique	0.403	Unique	yellow, gray, & white
*A. luteogularis*	Cuba	crown-giant	−0.49	Solid	orange/pink/yellow
*A. mestrei*	Cuba	trunk-ground	0.925	Unique	red, yellow, & white
*A. ophiolepis*	Cuba	grass-bush	−1.145	Solid	red
*A. paternus*	Cuba	twig	0.02	Unique	pink, yellow, & black
*A. porcatus*	Cuba	trunk-crown	−0.388	Solid	red
*A. pumilis*	Cuba	unique	−0.265	Solid	peach
*A. sagrei*	Cuba	trunk-ground	0.222	Marginal	red & yellow
*A. vandicus*	Cuba	grass-bush	0.199	Solid	yellow
*A. vermiculatus*	Cuba	unique	−1.407	–	–
*A. aliniger*	Hispaniola	trunk-crown	−0.402	Solid	green
*A. bahorucoensis*	Hispaniola	grass-bush	−1.217	Marginal	black & yellow
*A. brevirostris*	Hispaniola	trunk	−0.256	Basal/Solid/Marginal	yellow & red/orange/yellow/brown/gray/red/orange & yellow
*A. chlorocyanus*	Hispaniola	trunk-crown	−0.242	Lateral	gray & black, greenish-yellow & black
*A. christophei*	Hispaniola	unique	0.481	Striped	purple & pink
*A. distichus*	Hispaniola	trunk	−0.279	Solid/Marginal/Spotted/Basal	yellow, orange/red & yellow/red & yellow/red & yellow
*A. etheridgei*	Hispaniola	unique	−0.125	Solid	white/gray
*A. insolitus*	Hispaniola	twig	0.914	Solid	yellow/orange/brown
*A. longitibalis*	Hispaniola	trunk-ground	0.425	Solid	orange
*A. olssoni*	Hispaniola	grass-bush	0.579	Solid	orange
*A. semilineatus*	Hispaniola	grass-bush	0.361	Solid	white
*A. garmani*	Jamaica	crown-giant	0.086	Solid	yellow
*A. grahami*	Jamaica	trunk-crown	−0.024	Marginal	orange & yellow
*A. lineatopus*	Jamaica	trunk-ground	0.704	Marginal/Lateral/Unique/Spotted	orange & gray/gray & orange/green, white, orange/yellow & orange
*A. opalinus*	Jamaica	trunk-crown	0.466	Unique	red & yellow
*A. reconditus*	Jamaica	unique	0.685	Unique	orange & gray
*A. valencienni*	Jamaica	twig	0.46	Solid	purple
*A. cooki*	Puerto Rico	trunk-ground	0.16	Solid	orange
*A. cristatellus*	Puerto Rico	trunk-ground	0.036	Marginal/Solid/Basal	greenish-yellow & red/greenish-yellow/yellow
*A. cuvieri*	Puerto Rico	crown-giant	0.233	Solid	yellow
*A. evermanni*	Puerto Rico	trunk-crown	−0.127	Solid	yellow/green
*A. gundlachi*	Puerto Rico	trunk-ground	0.425	Solid	brown/yellow
*A. krugi*	Puerto Rico	grass-bush	0.225	Solid	yellow
*A. occultus*	Puerto Rico	twig	0.169	Lateral	gray & red
*A. poncensis*	Puerto Rico	grass-bush	−0.948	Solid	white/yellow
*A. pulchellus*	Puerto Rico	grass-bush	0.05	Basal	red & purple
*A. stratulus*	Puerto Rico	trunk-crown	0.338	Solid	red/orange

Abbreviations as follows: Community: C = Cuba, H = Hispaniola, J = Jamaica, and PR = Puerto Rico; Ecomorph:, CG = crown-giant, GB = grass-bush, TC = trunk-crown, TG = trunk-ground, TR = trunk, TW = twig; and U = unique. Dewlap size data comes from [16]. Species polymorphic for pattern (e.g., basal/solid/marginal) are indicated in the next to last column and the corresponding colors exhibited by each pattern for each species is indicated in the far right column. Polymorphism in color is similarly indicated (e.g., may have a solid pattern only but may vary in coloration, red/yellow/orange).

### Species Recognition Hypothesis

Of the four *Anolis* island communities, only one, from Puerto Rico, includes a species pair with identical dewlaps (Table 5). In the other three communities, the dewlap of every anole is unique within that community. These results are consistent with the Species Recognition hypothesis. However, our randomization test showed that this result is not unexpected given the distribution of dewlap colors and patterns among species (Table 6). Results were not qualitatively changed regardless of which dewlap morph of *A. cristatellus* was used.

**Table 5:** Data used to test the Species Recognition hypothesis.

Species	Community	Relative Size	Pattern	Color
*A. allogus*	Cuba	1.065	Striped	yellow & red
*A. alutaceus*	Cuba	0.396	Solid	yellow
*A. angusticeps*	Cuba	−0.206	Solid	pink
*A. homolechis*	Cuba	0.71	Solid	white/gray
*A. loysiana*	Cuba	0.863	Unique	yellow & red
*A. luteogularis*	Cuba	−0.49	Solid	orange/pink/yellow
*A. mestrei*	Cuba	0.925	Unique	red, yellow, & white
*A. porcatus*	Cuba	−0.388	Solid	red
*A. sagrei*	Cuba	0.222	Marginal	red & yellow
*A. vermiculatus*	Cuba	−1.407	–	–
*A. aliniger*	Hispaniola	−0.402	Solid	green
*A. chlorocyanus*	Hispaniola	−0.242	Lateral	gray & black, greenish-yellow & black
*A. christophei*	Hispaniola	0.481	Striped	purple & pink
*A. distichus*	Hispaniola	−0.279	Solid/Marginal/Spotted/Basal	yellow, orange/red & yellow/red & yellow/red & yellow
*A. etheridgei*	Hispaniola	−0.125	Solid	white/gray
*A. insolitus*	Hispaniola	0.914	Solid	yellow/orange/brown
*A. semilineatus*	Hispaniola	0.361	Solid	white
*A. garmani*	Jamaica	0.086	Solid	yellow
*A. grahami*	Jamaica	−0.024	Marginal	orange & yellow
*A. lineatopus*	Jamaica	0.704	Marginal/Lateral/Unique/Spotted	orange & gray/gray & orange/green, white, orange/yellow & orange
*A. opalinus*	Jamaica	0.466	Unique	red & yellow
*A. cristatellus*	Puerto Rico	0.036	Marginal/Solid/Basal	greenish-yellow & red/greenish-yellow/yellow
*A. cuvieri*	Puerto Rico	0.233	Solid	yellow
*A. evermanni*	Puerto Rico	−0.127	Solid	yellow/green
*A. gundlachi*	Puerto Rico	0.425	Solid	brown/yellow
*A. krugi*	Puerto Rico	0.225	Solid	yellow
*A. occultus*	Puerto Rico	0.169	Lateral	gray & red
*A. poncensis*	Puerto Rico	−0.948	Solid	white/yellow
*A. pulchellus*	Puerto Rico	0.05	Basal	red & purple
*A. stratulus*	Puerto Rico	0.338	Solid	red/orange

Not included in this analysis due to missing data were *A. cybotes* (Hispaniola), *and A. ricordii* (Hispaniola). Patterns and colors are as in Table 3.

**Table 6:** Number of pairs of identical dewlaps in the four anole communities, compared to communities assembled randomly without replacement from all anole species used in this study.

Island	Number of Included Species	Identical Pairs	P
Hispaniola	7	0	0.8651
Cuba	10	0	0.7313
Jamaica	5	0	0.9299
Puerto Rico A	8	1	0.9818
Puerto Rico B	8	1	0.9822
Puerto Rico C	8	2	0.9922
Puerto Rico D	8	1	0.9848

P-values represent the proportion of randomly assembled communities with the same or fewer identical pairs of species. The four results from Puerto Rico reflect the inclusion of four different dewlap forms for *A. cristatellus*.

## Discussion

**Figure 4:**
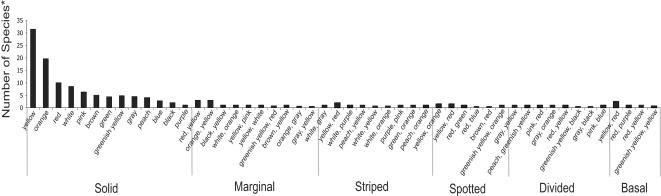
A histogram depicting the frequency of dewlap pattern and color combinations. *Data were weighted by the proportion of polymorphism exhibited by each species. For example, if a species exhibited four dewlap morphs and one was a solid yellow dewlap, a score of 0.25 was recorded for the solid yellow dewlap category.

Although dewlaps do exhibit impressive morphological diversity, our study shows that dewlaps can readily be placed into several discrete categories, with less than 10%of all possible combinations of color and pattern actually being observed among Caribbean anoles. Figure 4 shows the distribution of patterns and colors, and indicates the dominance of the Solid pattern and the color Yellow among Caribbean anoles. Of the Solid morphs, 30%are yellow and 21%are orange, with other colors decreasing dramatically in frequency. The functional significance for the predominance of Yellow and Orange dewlaps is unknown but is likely related to contrasting effects with background colors in their habitats [24]–[26], [46]. Interestingly, these colors are believed to be carotenoid-based [47], and carotenoids are generally obtained from the environment. Yet there is no evidence that diet plays any role in anole dewlap color and dewlap colors do not change seasonally (personal observation from all authors, no known citation reporting observed seasonal change).

### Phylogenetic Signal of Dewlap Configurations

Dewlap morphology is not phylogenetically informative. Dewlap patterns, colors, and sizes appear to be convergent features; that is, no relationship exists between how closely related two species are and how similar they are in any feature of the dewlap. This result parallels patterns of evolution in other aspects of anole morphology and ecology, which also show extensive convergence and lack of phylogenetic signal [18]–[19], [48].

### Ecomorph Convergence Hypothesis

For the Ecomorph Convergence hypothesis, the question we addressed was whether anoles living in similar structural habitats possessed similar dewlap configurations compared to those living in different habitats. We found that species using the same structural habitat (i.e., members of a specific ecomorph category) were no more similar in dewlap configuration than expected by chance. Losos and Chu [16] focused only on dewlap size and came to the same conclusion.

An issue in considering any negative result is whether the test employed had sufficient power to detect a real relationship, if one existed. In a comparable study with a smaller sample size (n = 21 species; no more than one representative of any ecomorph per island) using identical statistical methods was able to detect resoundingly significant ecomorph convergence for several sets of morphological characters, including body size, body shape, head shape, lamellae counts, and sexual size dimorphism [18], suggesting that these results are not merely a reflection of low statistical power. Consequently, we believe that the lack of significance in this case is unlikely to be a result of lack of power.

Our results thus indicate that, unlike other morphological characteristics, dewlap configuration is not related to structural habitat use. In other words, dewlap features have clearly not evolved in concert with the suite of characteristics that define the anole ecomorphs [cf. 21], [49].

In retrospect, this result is not surprising. Members of the same ecomorph category are often found in very different light environments, ranging from dark forest to open sunlight [27]; [49]. Thus, the light environment differs among species in the same structural habitat, and, as would be expected, dewlap configurations differ as well. These results thus agree with studies that have shown that species' dewlaps have evolved to optimize their visibility/detectability in the habitats in which the species occur [24]–[25], [27]–[28]. Once data become available regarding specific light environments for each species perhaps this hypothesis could be investigated again to determine if ecomorphs in similar light environments have converged in dewlap morphologies.

### Species Recognition Hypothesis

The lack of support for the Species Recognition hypothesis was surprising. The results were not only not significant, but nowhere near significant (p-values ranged from 0.87 to 0.99). Support for the function of color in species recognition has been demonstrated in lab experiments between species pairs [e.g.], [ 29], [ 50–51], and, while not tested directly, several other studies have indicated support for this hypothesis [24]–[26], [Bibr pone.0000274-Leal3] [28; 30]. Rand and Williams [17] applied this hypothesis to larger communities of anoles and proposed it as a general explanation for dewlap diversity.

Our study is the first to address this question across a broad sampling of anoles while analyzing the data within a phylogenetic context, and we find that, although the data within each community are consistent with the Species Recognition hypothesis, they do not provide statistical corroboration. In other words, the reason we fail to find support for this hypothesis is not because such a pattern does not exist, but simply because such a pattern is not unlikely. Given the great variety of dewlap configurations that exist across each island, our findings indicate that even communities in which no dewlaps co-occur is rather likely to occur by chance. For this reason, enormous sample sizes would probably be needed to distinguish real patterns from random expectations. Consequently, we conclude that comparative studies such as this are extremely unlikely to provide evidence that sympatric species do not share dewlap configurations as a result of species recognition problems. We suggest, instead, that workers interested in such questions focus on experimental approaches to assess the role of dewlap configuration in species recognition [29], [50].

Our sampling scheme involved choosing species-rich communities as exemplars for each island in the Greater Antilles. However, great variation exists in community composition across each of these islands. For example, some species are widespread, whereas many others have more local distributions (see range maps in [12]). Moreover, some ecomorphs are absent from some areas, for reasons that are often unclear [52]. One alternative approach that would be worth further investigation would be to include a geographic component in studies of dewlap diversity. It is possible, for example, that the dewlaps of widespread species have evolved to be particularly distinct from other species with which they co-occur widely, as opposed to species which they overlap only in a portion of their range. Moreover, it is also possible that species exhibit geographic variation in dewlap configuration that results from the different ensemble of species with which they coexist at different localities. Such a study would require much more detailed information than is currently available, but would be well worth the effort.

### Future Directions

Studies on dewlap evolution could be further developed in several other ways. First, we classified colors subjectively because literature descriptions and personal observations did not follow standardized color charts [e.g., 38] or measure dewlap reflectance [sensu 25 and references therein], [ 27–28]. Our approach has the effect of underestimating the amount of variation that exists in dewlap color, both by lumping color variation into several categories and by overlooking the existence of ultraviolet reflectance, which has been reported in some anoles [46]. From the studies of Fleishman and colleagues it is known that the vision of some (and perhaps all) anole vision extends into the ultraviolet and that some anole dewlaps reflect UV [24]–[28], [46].

In addition, our preliminary approach to color quantification could be improved by collecting reflectance data and would likely reveal even more variation. But given the large number of species, and the inaccessibility of some of them, such studies are not likely to be possible in the near future. In any case, our approach here is conservative because recognizing greater variation in dewlap color would lead to even *less* support for the hypotheses we investigated. In other words, our study found substantial variation in dewlap morphology and the hypotheses we tested lacked significant support because so much variation exists; increased variation would only render results less significant (where that is possible).

One alternative explanation for dewlap diversity may be sexual selection. Fitch and Hillis [15] presented evidence that suggests sexual selection could explain, in part, an association between dewlap morphology, habitat, and breeding season length in mainland anoles. They found that seven anoles in seasonal habitats had large, brightly colored dewlaps relative to ten species living in wetter, less seasonal habitats. They hypothesized that this might be due to the shorter breeding season in seasonal habitats in which competition for mates might be more intense. While sample sizes were small and not analyzed within a phylogenetic context, their study reveals an interesting pattern, but is limited in its ability to explain dewlap configuration diversity because it only shows an association between bright (vs. dull) coloration, size (large vs. small), and habitat (dry vs. wet). More direct measures of the effect of dewlap color on male-male competition and female mate choice are needed to assess the sexual selection hypothesis.

In this study we have only compared the dewlaps of male anoles, but the females of some species also possess dewlaps [12]. Explaining variation in female dewlaps—both whether they are present and, if so, how they compare to the dewlap configurations of their male conspecifics—would be a topic of considerable interest. Our review of the literature indicates that the females of 20 species of Caribbean anoles possess dewlaps. Of these, approximately half exhibit dewlaps identical to those of their male counterparts, whereas the others differ in some way, primarily in color, but sometimes also in pattern. All 20 of the species in which females possess dewlaps are arboreal (e.g., crown-giant, trunk-crown, or twig).

Why some females possess dewlaps and others do not has never been examined. One possibility is that the presence of a dewlap in females may correlate with the degree of female territoriality. Perhaps the social system of arboreal anoles is different from more terrestrial species, but this topic has not yet been studied with respect to females. Another possibility is that sexual selection pressures are different in different habitats and have led to reduced sexual dimorphism in particular instances. Two studies [22]–[23] examined size and shape dimorphism in anoles, but our comparison of these studies with our review of female dewlap information suggests that neither size nor shape dimorphism correspond with female dewlap possession (Nicholson et al., unpubl.). The functional significance of female dewlap possession therefore remains an interesting subject for future research.

A plethora of studies has demonstrated the importance of the dewlap for anole behavior [e.g.], [ 4], [ 13]–[15], [ 24]–[25], [ 50], [ 53]–[55], yet several recent studies have failed to show that experimental disabling of the dewlap has any effect on territory ownership or mating rate, at least in one species, *A. sagrei* [56]–[58]. These results are surprising, although they involve only one species and have been of short duration with relatively small sample sizes. Further work is needed to investigate the functional role of dewlaps in extant species.

This study provides a characterization of dewlap diversity for Caribbean anoles and serves as a foundation for other studies seeking to address questions of dewlap evolution. Dewlap configurations are diverse, but clearly much remains to be learned regarding *Anolis* dewlap evolution and function. Numerous aspects of signal communication and evolution remain to be explained, including the relationship between dewlap configurations and reflectance, and how these characters are adapted to suit their backgrounds.

## References

[1] Espmark Y, Amundsen T, Rosenqvist G (2000). Animal signals: signaling and signal design in animal communication..

[2] erhardt HC, Huber F (2002). Acoustic communication in insects and anurans..

[3] Greenfield MD (2002). Signalers and receivers: mechanisms and evolution of arthropod communication..

[4] Jenssen TA (1977). Evolution of Anoline lizard display behavior.. Am Zool.

[5] Ord TJ, Blumstein DT (2002). Size constraints and the evolution of display complexity: why do large lizards have simple displays?. Biol J Linn Soc..

[6] Ord TJ, Blumstein DT, Evans CS (2002). Ecology and signal evolution in lizards.. Biol J Linn Soc.

[7] Dawkins MS (1993). Are there general principles of signal design?. Phil Trans Roy Soc Lond B.

[8] Endler JA (1993). Some general comments on the evolution and design of animal communication systems.. Phil Trans Roy Soc Lond B.

[9] Martins EP, Labra A, Hallloy M, Thompson JR (2004). Large-scale patterns of signal evolution: an interspecific study of *Liolaemus* lizard headbob displays.. An Behav.

[10] Bels VL (1990). The mechanism of dewlap extension in *Anolis carolinensis* (Reptilia: Iguanidae) with histological analysis of the hyoid apparatus.. J Morph.

[11] Font E, Rome LC (1990). Functional morphology of dewlap extension in the lizard *Anolis equestris* (Iguanidae).. J Morph.

[12] Schwartz A, Henderson RW (1991). Amphibians and reptiles of the West Indies: descriptions, distributions, and natural history..

[13] Williams EE, Rand AS (1977). Species recognition, dewlap function and faunal size.. Am Zool.

[14] Leal M, Rodríguez-Robles JA (1997). Anti-predator responses of the Puerto Rican giant anole, *Anolis cuvieri* (Sauria: Polychrotidae).. Biotrop.

[15] Fitch HS, Hillis DM (1984). The *Anolis* dewlap: interspecific variability and morphological associations with habitat.. Copeia.

[16] Losos JB, Chu L (1998). Examination of factors potentially affecting dewlap size in Caribbean anoles.. Copeia.

[17] Rand AS, Williams EE (1970). An estimation of redundancy and information content of anole dewlaps.. Am Nat.

[18] Harmon LJ, Kolbe JJ, Cheverud JM, Losos JB (2005). Convergence and the multidimensional niche.. Evol.

[19] Losos JB, Jackman TR, Larson A, de Queiroz K, Rodriguez-Schettino L (1998). Contingency and determinism in replicated adaptive radiations of island lizards.. Science.

[20] Nicholson KE, Glor RE, Kolbe JJ, Larson A, Hedges SB (2005). Mainland colonization by island lizards.. J Biogeo.

[21] Beuttell K, Losos JB (1999). Ecological morphology of Caribbean anoles.. Herp Mono.

[22] Butler MA, Schoener TW, Losos JB (2000). The relationship between sexual size dimorphism and habitat use in Greater Antillean *Anolis* lizards.. Evol.

[23] Butler MA, Losos JB (2002). Multivariate sexual dimorphism, sexual selection, and adaptation in Greater Antillean *Anolis* lizards.. Ecol Mono.

[24] Fleishman LJ (1992). The influence of the sensory system and the environment on motion patterns in the visual displays of Anoline lizards and other vertebrates.. Am Nat (suppl.).

[25] Fleishman LJ, Espmark Y, Amundsen T, Rosenqvist G (2000). Signal function, signal efficiency, and the evolution of Anoline lizard dewlap color.. Animal Signals: Signaling and Signal Design in Animal Communication.

[26] Fleishman LJ, Persons J (2001). The influence of stimulus and background colour on signal visibility in the lizard *Anolis cristatellus*.. J Exp Biol.

[27] Leal M, Fleishman LJ (2002). Evidence for habitat partitioning based on adaptation to environmental light in a pair of sympatric lizard species.. Proc R Soc Lond B.

[pone.0000274-Leal3] Leal M, Fleishman LJ (2004). Differences in visual signal design and detectability between allopatric populations of *Anolis* lizards.. Am Nat.

[29] Losos JB (1985). An experimental demonstration of the species-recognition role of *Anolis* dewlap color.. Copeia.

[30] Persons MH, Fleishman LJ, Frye MA, Stimphil ME (1999). Sensory response patterns and the evolution of visual signal design in Anoline lizards.. J Comp Physiol A.

[31] Jackman TR, Larson A, de Queiroz K, Losos JB (1999). Phylogenetic relationships and tempo of early diversification in *Anolis* lizards.. Syst Biol.

[32] Nicholson KE (2002). Phylogenetic analysis and a test of the current infrageneric classification of *Norops* (beta *Anolis*).. Herp Mono.

[33] Poe S (2004). Phylogeny of anoles.. Herp Mono.

[34] Ashton RE, Ashton PS (1991). Handbook of Reptiles and Amphibians of Florida: Part Two: Lizards, Turtles, and Crocodilians..

[35] Powell R, Henderson RW (1996). Contributions to West Indian Herpetology: A tribute to Albert Schwartz..

[36] Rivero JA (1978). The Amphibians and Reptiles of Puerto Rico..

[37] Rodriguez-Schettino LR (1999). The iguanid lizards of Cuba..

[38] Smithe FB (1981). Naturalist's color guide..

[39] Sanderson MJ (2003). r8s: inferring absolute rates of molecular evolution and divergence times in the absence of a molecular clock.. Bioinformatics.

[40] Rambaut A, Charleston M (2001). TreeEdit v. 1.08..

[41] Böhning-Gaese KM, Schuda D, Helbig AJ (2003). Weak phylogenetic effects on ecological niches of *Sylvia* warblers.. J Evol Biol.

[42] Casgrain P (2001). Permute! Vers. 3.4 alpha..

[43] Williams EE (1972). Origin of faunas: evolution of lizard congeners in a complex island fauna: a trial analysis.. Evol Biol.

[44] Smouse PE, Long JC, Sokal RR (1986). Multiple regression and correlation extensions of the Mantel test of matrix correspondence.. Syst Zool.

[45] Kolbe JJ, Glor RE, Rodriguez-Schettino L, Lara AC, Larson A (2004). Genetic variation increases during biological invasion of a Cuban lizard.. Nature.

[46] Fleishman LJ, Loew ER, Leal M (1993). Ultraviolet vision in lizards.. Nature.

[47] Cooper WE, Greenberg N, Gans C, Crews D (1992). Reptilian coloration and behavior.. Biology of the Reptilia: Hormones, Brain, and Behavior Series.

[48] Losos JB, Dieckmann U, Doebeli M, Metz JAJ, Tautz D (2004). Adaptation and speciation in Greater Antillean anoles.. Adaptive Speciation.

[49] Williams EE, Huey RB, Pianka ER, Schoener TW (1983). Ecomorphs, faunas, island size, and diverse endpoints in island radiations of *Anolis*.. Lizard Ecology: studies of a model organism..

[50] Macedonia JM, Stamps JA (1994). Species recognition in the lizard, *Anolis grahami* (Reptilia, Iguanidae): Evidence from video playbacks of conspecific and heterospecific displays.. Ethol.

[51] Macedonia JM, Evans CS, Losos JB (1994). Male *Anolis* lizards discriminate video-recorded conspecific and heterospecific displays.. An Behav.

[52] Moermond TC (1979). Habitat constraints on the behavior, morphology, and community structure of *Anolis* lizards.. Ecol.

[53] Echelle AA, Echelle AF, Fitch HS (1971). A comparative analysis of aggressive display in nine species of Costa Rican *Anolis*.. Herp.

[54] Fitch HS, Henderson RW (1987). Ecological and ethological parameters in *Anolis bahorucoensis*, a species having rudimentary development of the dewlap.. Amph-Rept.

[55] Rodrigues MT, Xavier V, Skuk G, Pavan D (2002). New specimens of *Anolis phyllorhinus* (Squamata, Polychrotidae): the first female of the species and of proboscid anoles.. Pap Avul Zool, São Paulo.

[56] Tokarz RR (2002). An experimental test of the importance of the dewlap in male mating success in the lizard *Anolis sagrei*.. Herp.

[57] Tokarz RR, Paterson AV, McMann S (2003). Laboratory and field test of the functional significance of the male's dewlap in the lizard *Anolis sagrei*.. Copeia.

[58] Tokarz RR, Paterson AV, McMann S (2005). Importance of dewlap display in male mating success in free-ranging brown anoles (*Anolis sagrei*).. J Herp.

